# Development of a flow-fluorescence *in situ* hybridization protocol for the analysis of microbial communities in anaerobic fermentation liquor

**DOI:** 10.1186/1471-2180-13-278

**Published:** 2013-12-04

**Authors:** Edith Nettmann, Antje Fröhling, Kathrin Heeg, Michael Klocke, Oliver Schlüter, Jan Mumme

**Affiliations:** 1 APECS junior research group, Leibniz Institute for Agricultural Engineering, Max-Eyth-Allee 100, 14469 Potsdam, Germany; 2 Institute of Environmental Engineering, Ruhr University Bochum, Universitätsstrasse 150, 44780 Bochum, Germany; 3 Quality and Safety of Food and Feed, Leibniz Institute for Agricultural Engineering, Max-Eyth-Allee 100, 14469 Potsdam, Germany; 4 Department Bioengineering, Leibniz Institute for Agricultural Engineering, Max-Eyth-Allee 100, 14469 Potsdam, Germany; 5 Faculty of Process Sciences, Institute of Technical Environmental Protection, Environmental Microbiology, Technical University Berlin, Ernst-Reuter-Platz 1, 10587 Berlin, Germany

**Keywords:** Flow cytometry, Fluorescence *in situ* hybridization, Flow-FISH, Biogas reactor, Upflow anaerobic solid state (UASS) reactor, Anaerobic digestion

## Abstract

**Background:**

The production of bio-methane from renewable raw material is of high interest because of the increasing scarcity of fossil fuels. The process of biomethanation is based on the inter- and intraspecific metabolic activity of a highly diverse and dynamic microbial community. The community structure of the microbial biocenosis varies between different biogas reactors and the knowledge about these microbial communities is still fragmentary. However, up to now no approaches are available allowing a fast and reliable access to the microbial community structure. Hence, the aim of this study was to originate a Flow-FISH protocol, namely a combination of flow cytometry and fluorescence *in situ* hybridization, for the analysis of the metabolically active microorganisms in biogas reactor samples. With respect to the heterogenic texture of biogas reactor samples and to collect all cells including those of cell aggregates and biofilms the development of a preceding purification procedure was indispensable.

**Results:**

Six different purification procedures with in total 29 modifications were tested. The optimized purification procedure combines the use of the detergent sodium hexametaphosphate with ultrasonic treatment and a final filtration step. By this treatment, the detachment of microbial cells from particles as well as the disbandment of cell aggregates was obtained at minimized cell loss. A Flow-FISH protocol was developed avoiding dehydration and minimizing centrifugation steps. In the exemplary application of this protocol on pure cultures as well as biogas reactor samples high hybridization rates were achieved for commonly established domain specific oligonucleotide probes enabling the specific detection of metabolically active bacteria and archaea. Cross hybridization and autofluorescence effects could be excluded by the use of a nonsense probe and negative controls, respectively.

**Conclusions:**

The approach described in this study enables for the first time the analysis of the metabolically active fraction of the microbial communities within biogas reactors by Flow-FISH.

## Background

The foreseeable scarcity of fossil fuels promoted the development of innovative techniques for the generation of alternative energies in the last years. In this case, the utilization of renewable raw materials such as agricultural biomass or organic wastes represents an important cornerstone for the production of renewable energy.

In the last years, the investigation of microbial biocenoses responsible in biogas reactors for the production of methane-rich biogas became a matter of particular interest. Several studies led to the conclusion that a uniform microbial community in biogas reactors does not exist and, in addition of it, there are still gaps of knowledge about the microflora in this environment [1-5]. To overcome this lack of knowledge the establishment of a fast and reproducible analytical tool for the specific detection of the metabolically active microorganisms in this environment is of high relevance.

Beside gene based quantification techniques such as quantitative real-time PCR, the hybridization of microbial cells with 16S ribosomal RNA (16S rRNA) targeting fluorescently labeled oligonucleotides (fluorescent *in situ* hybridization, FISH) and a subsequent microscopic cell counting is the method of choice for the quantification of microorganisms in environmental samples [6,7]. The benefit of this technique is the cell based quantification of microorganisms at different taxonomic levels depending on the degree of conservation of the probe target sequence [8].

However, some potential pitfalls of FISH are well known and should be noted [9,10]. One of the most critical steps is the fixation of samples. The fixative saves the cell morphology while simultaneously the cell membrane is permeabilized for the labeled oligonucleotides. In addition, this step prevents cell lysis during hybridization and subsequent storage. Because of different characteristics of the cell membrane of Gram-negative and Gram-positive cells, different fixatives have to be used [11]. Whereas fixation with cross-linking agent formaldehyde or paraformaldehyde is strengthen the cell wall of Gram-negative prokaryotes, the cell wall of Gram-positive bacteria will be damaged by these fixatives. Therefore, it is recommended to fix Gram-positive cells with ethanol.

Besides fixation, the metabolic activity state of the analyzed cells has also a high impact on the FISH results because most common FISH probes target the 16S rRNA molecules in prokaryotic cells. The number of ribosomes is strongly depending on the metabolic activity of the cell. Prokaryotic cells with low metabolic activity or in a dormant state may have a low content of ribosomes and in consequence a low content of probe targets which results in hardly proven fluorescence signals [6,7,12,13]. Nevertheless, for the analysis of the microbial community of biogas reactors the detection of active cells is of special interest because these cells are responsible for biogas generation from biomass.

The conventional FISH approach is very time-consuming due to the essential number of technical and biological replicates that have to be performed. As an alternative method, flow cytometry allows high-throughput quantification and simultaneously the phenotypic separation of cell populations based on differences in surface characters of single cells [12,14]. Recently, flow cytometry was successfully applied for the analyses of the microbial community structure in different environmental samples to generate cytometric fingerprints using DNA-intercalating dyes such as 4’,6-diamidino-2-phenylindole (DAPI) [15-17].

However, staining with DNA-intercalating fluorochromes may provide information on the amount of microbial cells in a given sample but not on their taxonomic identity [12]. This lack can be overcome by the combination of flow cytometry and FISH. This approach is called Flow-FISH and was described for the first time by Rufer and co-workers (1998) [18] within the scope of the analysis of human lymphocytes. In respect to the analysis of microbial cells the Flow-FISH technique was firstly applied by Friedrich and Lenke (2006) [19]. Since then, the Flow-FISH has already been applied successfully for the analysis of pure cultures [20] as well as the analysis of mixed microbial populations [12]. Furthermore, this technique was used for the monitoring of specific clostridial cells in an anaerobic semi-solid bio-hydrogen producing system [21]. In addition, Flow-FISH could be an innovative technique for microbiological analyses of biogas reactors samples.

However, the Flow-FISH based analysis of microbial communities in biogas reactors is strongly hampered by the high heterogeneity of the sample material due to the presence of organic (e.g. plant fibers) and inorganic particles which cause high background fluorescence signals. Moreover, most of the process relevant microorganisms adhere on these particles and form complex biofilms or form dense cell clusters like *Methanosaeta* spp. and *Methanosarcina* spp. [22,23]. This hampers any cell counting attempt by microscopy as well as flow cytometry. In addition, some of these cell associations can reach a thickness that inhibits the penetration of FISH probes into deeper layers of cell clusters. In consequence, only the surface cells are hybridized with FISH probes and are detectable by Flow-FISH. Hence, samples from this environment have to be pretreated to purify and to isolate all microbial cells of the whole biogas reactor biocenosis. Despite the number of different pretreatment approaches developed for a variety of samples of different environmental origins [24-28], up to now no procedures are published for the purification of samples from biogas reactors leading to preparations suited for the measurement of the microbial community by Flow-FISH.

To overcome these technical limitations, the aim of this study was to establish a high-throughput technique for the detection and the quantification of process relevant, active microorganisms in anaerobic digestion using the process liquor of an upflow anaerobic solid-state (UASS) biogas reactor as test material [29]. Therefore, a purification technique was primarily optimized to fulfill the following requirements: (1) detachment of cells from organic and inorganic particles, (2) disbandment of cell aggregates, (3) no or low cell loss, and (4) a rapid implementation. Furthermore, a modified Flow-FISH protocol based on different already published protocols [12,20,30] was developed and tested regarding following influencing parameters: (1) type of fixative used for cell fixation directly after sampling, (2) possible cell losses by centrifugation during FISH procedure, and (3) cell activity.

## Results and discussion

### Optimization of the purification technique

The application of flow cytometry for the analysis of the microbial community in biogas reactors requires previous sample purification due to its high content of organic and inorganic particles and the presence of huge cell aggregates and biofilms. The capillary within the flow cytometer could clog due to such large particles. Moreover, the microbes bound in aggregates and biofilms are hardly detectable and countable with the Flow-FISH.

**Figure 1:**
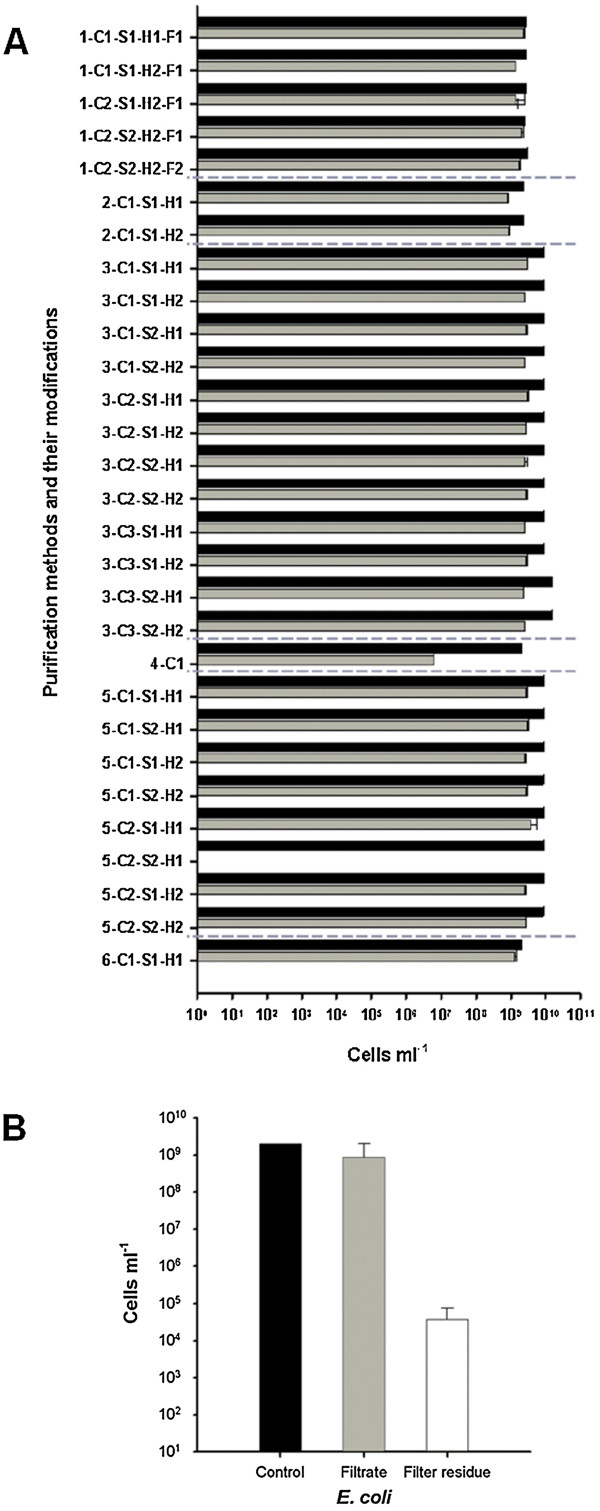
**Influencing factors of purifications treatments on cell counts determined by Coulter Counter. (A)** Cell counts for *E. coli* cultures before (black bars) and after (gray bars) purification procedures. Denomination of procedures is according to Table 1. Error bars resulted from nine different measurements. **(B)** Influence of filtration: Cell counts of *E. coli* purified with procedure 1-C2-S2-H1-F2 prior to vacuum filtration with a 12–15 μm filter (black bar), after filtration (grey bar), and cell counts of residues on the filter (white bar). Error bars resulted from three different measurements.

In this study, six purification procedures with in total 29 modifications were tested (Table 1). These six purification strategies are based on the use of a detergent to dissolve cell aggregates and to detach cells from different surfaces in soils [24-26,28] or turbid seawater [27]. A current method to increase the effect of detergent is the ultrasonic treatment [31] and homogenization of the sample with a dispersion unit [26]. The concentration of the used detergent and the settings of ultrasound and homogenization should be adjusted because these treatments can also destroy the cell wall of microbes. Therefore, cell numbers were determined by Coulter Counter system in order to control cell losses caused by sample pretreatment. However, due to the heterogeneity of sample material derived from biogas reactors a control of cell counts with the Coulter Counter system before and after purification procedures was not feasible. Thus, a pure *E. coli* culture was used to control possible cell losses during the different procedures (Figure 1A).

**Table 1:** Purification procedures and modifications

**Procedures**	**References**	**Detergents**	**Detergent concentrations (C)**	**Ultrasound treatment (S)**^**1)**^	**Homogenization (H)**^**2)**^	**Filtration (F)**
1	S.B. Singh-Verma (1968), LR. Bakken (1985)	Sodium hexametaphosphate	C1) 0,2% (w/v)	S1) 40 W, 60 sec, 5 impulses/sec (different repetitions)	H1) none	F1 none
			C2) 0,5% (w/v)	S2) 65 W, 60 sec, 5 impulses/sec (different repetitions)	H2) 60 sec, speed 5 (different repetitions)	F2) 12–15 μm filter
2	S.B. Singh-Verma (1968), LR. Bakken (1985)	Bromhexine hydrochloride	C1) 0,2% (w/v)	S1) 40 W, 60 sec + 65 W, 60 sec, 5 impulses/sec	H1) none	n.a.
					H2) 2× 60 sec, speed 5	
3	W.B. Yoon and R.A. Rosson (1990)	Tween	C1) 5 μg/ml	S1) 15 W, 30 sec, 5 impulses/sec	H1) none	n.a.
			C2) 10 μg/ml	S2) 35 W, 30 sec, 5 impulses/sec	H2) 5 min, speed 5	
			C3) 25 μg/ml			
4	E.L Schmidt (1974)	Tween 80 + 0.007 g ml^-1^ flocculation reagent (Ca (OH)_2_: MgCO_3_ (2:5))	C1) 25 μl/ml	n.a.	n.a.	n.a.
5	O. Resina-Pelfort et al. (2003)	Triton X-100	C1) 10 μg/ml	S1) 35 W, 30 sec, 5 impulses/sec	H1) none	n.a.
			C2) 20 μg/ml	S2) 45 W, 30 sec, 5 impulses/sec	H2) 5 min, speed 5	
6	L R. Bakken (1985)	Sodium pyrophosphate	C1) 0,2% (w/v)	S1 3× 40 W, 60 sec, 5 impulses/sec	H1) 3× 60 sec, speed 5	n.a.

n.a. = not applied.

^1)^using the Sonoplus GW2070 (Bandelin, Germany).

^2)^using the dispersion unit VDI12 for 0.1 - 5.0 ml volumes (VWR, Germany).

C1-3, H1-2, S1-2 and F1-2 indicate variations of the original protocols tested for their eligibility on samples from pure cultures and the UASS biogas reactor.

With exception of procedure 4-C1 and 5-C2-S2-H1 (see Table 1 for details) the cell losses of control samples during purification were marginal. Best results were obtained with procedure 1, using sodium hexametaphosphate as detergent, and procedure 6, with sodium pyrophosphate as detergent (Figure 1A). To determine the presence and the size of cell aggregates as well as cells attached to debris, the differentially treated samples were examined visually by fluorescence microscopy (Table 2).

**Table 2:** Evaluation of purification procedures and their modifications by fluorescence microscopy

**Procedure**	**Cell aggregates present**	**Maximum cell aggregate size**^**1)**^	**Abiotic particles present**	**Abiotic particles covered with cells**
1-C1-S1-H1-F1	yes	+++	yes	no
1-C1-S1-H2-F1	yes	++	yes	no
1-C2-S1-H1-F1	yes	++	yes	no
1-C2-S1-H2-F1	yes	+	yes	no
1-C2-S2-H1-F1	no	-	yes	no
*1-C2-S2-H1-F2*	*no*	*-*	*no*	*no*
2-C1-S1-H1	yes	+++	yes	yes
2-C1-S1-H2	yes	+++	yes	yes
3-C1-S1-H1	yes	+++	yes	yes
3-C1-S1-H2	yes	++	yes	yes
3-C1-S2-H1	yes	++	yes	yes
3-C1-S2-H2	yes	+	yes	yes
3-C2-S1-H1	yes	+++	yes	yes
3-C2-S1-H2	yes	++	yes	yes
3-C2-S2-H1	yes	++	yes	yes
3-C2-S2-H2	yes	++	yes	yes
3-C3-S1-H1	yes	++	yes	yes
3C3-S1-H2	yes	++	yes	yes
3-C3-S2-H1	yes	++	yes	yes
3-C3-S2-H2	yes	+	yes	yes
4-C1-H1	yes	+++	yes	yes
5-C1-S1-H1	yes	+++	yes	yes
5-C1-S2-H1	yes	+++	yes	yes
5-C1-S1-H2	yes	++	yes	yes
5-C1-S2-H2	yes	++	yes	yes
5-C2-S1-H1	yes	+++	yes	yes
5-C2-S2-H1	yes	+++	yes	yes
5-C2-S1-H2	yes	++	yes	yes
5-C2-S2-H2	yes	+	yes	yes
6-C1-S1-H1	yes	++	yes	yes

^1)^ +++ = ≥ 52 μm^2^; ++ = ≥ 24 μm^2^; + = ≥ 6 μm^2^; - = no cell aggregates. The size of cell aggregates was determined by microscopic field analyses using an ocular micrometer at 630× magnification. One field covered an area of 5.76 μm^2^.

Denomination of procedures is according to Table 1. The optimal combination is given in italics.

Overall, the purification procedure 1 using the detergent sodium hexametaphosphate provided the best results concerning the disbandment of cell aggregates and biofilms and the elimination of organic and inorganic particles from the biogas reactor samples with a minimal cell loss during purification procedure. The final power of ultrasonic treatment and the sodium hexametaphosphate concentration for procedure 1 without filtration (1-C2-S2-H1-F1) was 60 W (60 sec) and 0.5% (w/v), respectively, which finally resulted in an almost complete recovery of cells from particles and disbandment of cell aggregates (Table 2).

**Figure 2:**
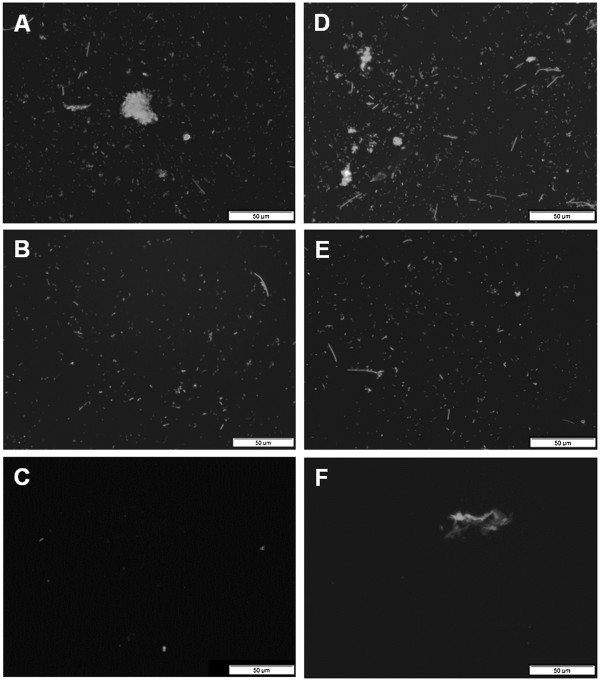
**Microscopic verification of purification procedure 1-C2-S2-H1-F2 at 400**× **magnification. A-C)** Microscopic image of UASS-1 reactor. **D-F)** Microscopic image of UASS-2 reactor at different times of sampling. Images **A** and **D** represents samples before purification procedure, images **B** and **E** represent samples after purification procedure whereas images **C** and **F** show residues on the filter. All samples were diluted 500-fold. Cells were stained with DAPI. Microscopic images were generated using a Nikon Optiphot-2 microscope (Nikon, Duesseldorf, Germany) and a DAPI AMCA filter tube. Scale bar equals 50 μm.

After repeated detergent and ultrasound treatment for a maximum of five times all supernatants were pooled and centrifuged at 8,000 × *g* for 20 min to collect all cells in a pellet and subsequently re-suspended in one fold concentrated phosphate buffered saline (1× PBS). A microscopic validation of this cell suspension showed a contamination with plant fibers and other inorganic particles which were free of cells, but made the samples unusable for analysis by Flow-FISH. Therefore a final vacuum filtration using a filter with a pore size of 12-15 μm was conducted. The cell loss resulting from filtration seemed to be negligible as the control experiment using *E. coli* cultures treated with procedure 1-C2-S2-H1-F2 revealed (Figure 1B). Figure 2 shows exemplary microscopic images of the application of purification procedure 1-C2-S2-H1-F2 using two different samples from the UASS biogas reactor (UASS-1 and UASS-2). The microbial cells were stained with DNA-binding fluorescence dye 4’,6-diamidino-2-phenylindole (DAPI). Before purification, small and large particles covered with cells as well as cell aggregates were observed in the UASS samples (Figure 2A, D). After application of purification procedure 1-C2-S2-H1-F2, these large particles were no longer present in the samples (Figure 2B, E). The microscopic analysis of residues on the filter (Figure 2C, F) resulted in only few single cells and cell free particles. This confirmed the results of purification treatment shown in Figure 2 (B, C).

**Figure 3:**
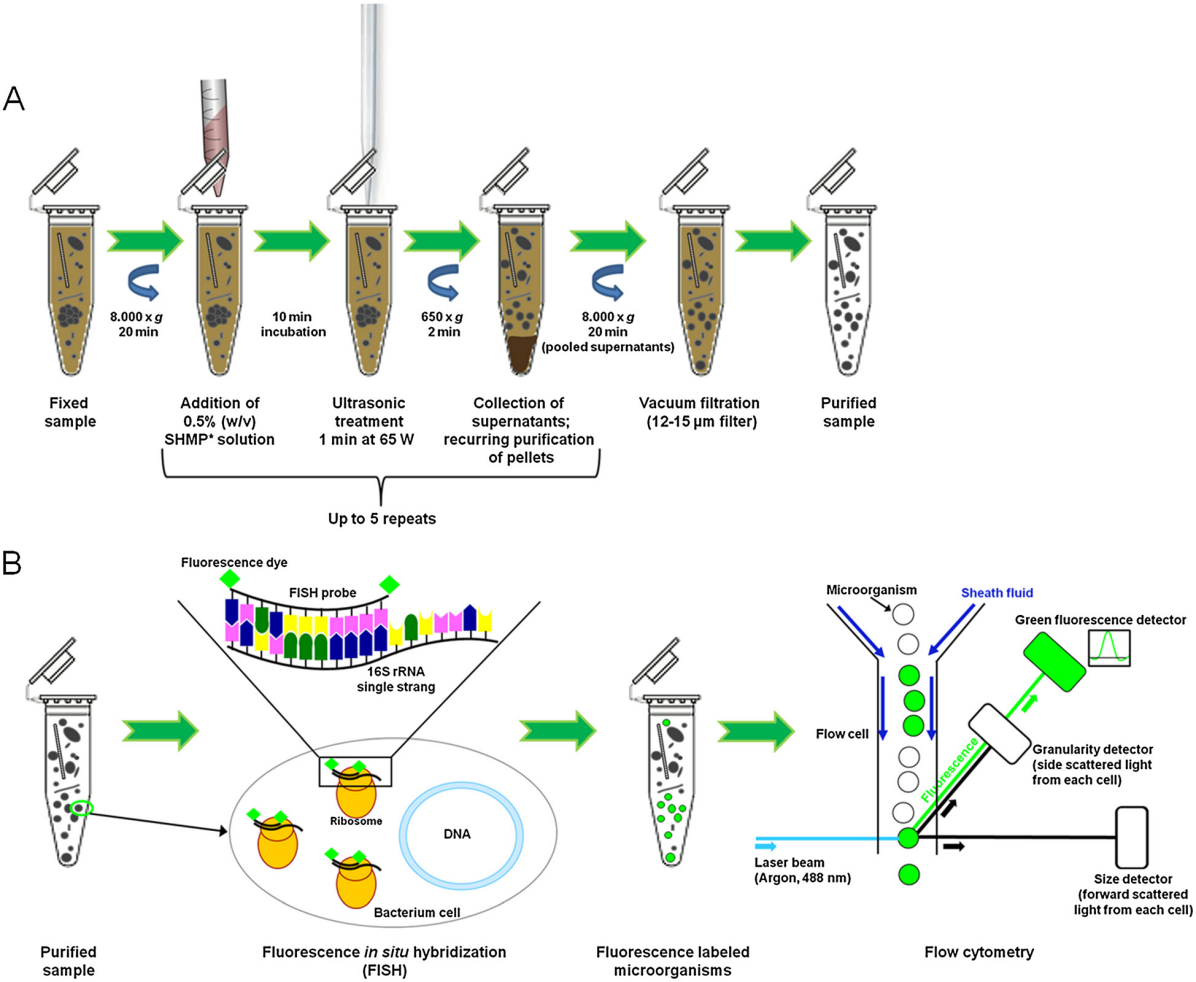
**Schematic figure illustrating the design and the principles of Flow-FISH protocol established in this study. (A)** Single steps of optimized purification procedure 1-C2-S2-H1-F2. **(B)** The purified sample is used for Flow-FISH, a combination of fluorescence *in situ* hybridization (FISH) and a subsequent analysis by flow cytometry. During FISH the 16S rRNA molecules of target microorganisms are hybridized with fluorescence labeled oligonucleotides (FISH probes). Samples with fluorescence labeled microorganisms are analyzed by flow cytometer. In the flow cell fluorescently labeled particles are delivered in single file to pass a focused light beam. The fluorescence emission of labeled cells is detected simultaneously with the detection of the scattered light of particles in two directions representing the cell size and granularity. *SHMP = sodium hexametaphosphate.

In conclusion, the procedure 1-C2-S2-H1-F2 using 0.5% sodium hexametaphosphate as detergent in combination of 60 W ultrasound treatment for 60 sec and a final filtration showed the best results and was subsequently used for the pretreatment of UASS biogas reactor samples for microbial analysis by Flow-FISH. However, it must be noted that, depending on the actual grade of heterogeneity of the biogas reactor sample, the optimized purification procedure will require some time. Figure 3 illustrates the different steps of the optimized purification procedure established in this study and the principle of the Flow-FISH technique.

### Establishment of a Flow-FISH protocol

Flow cytometry is a rapid high-throughput technique for the examination of microbial cells and a process in which characteristics of single cells are measured in a fluid stream [32]. In combination with FISH technique, so called Flow-FISH, the taxonomic identification of single microorganisms in microbial community and the cell quantification will be feasible simultaneously.

**Figure 4:**
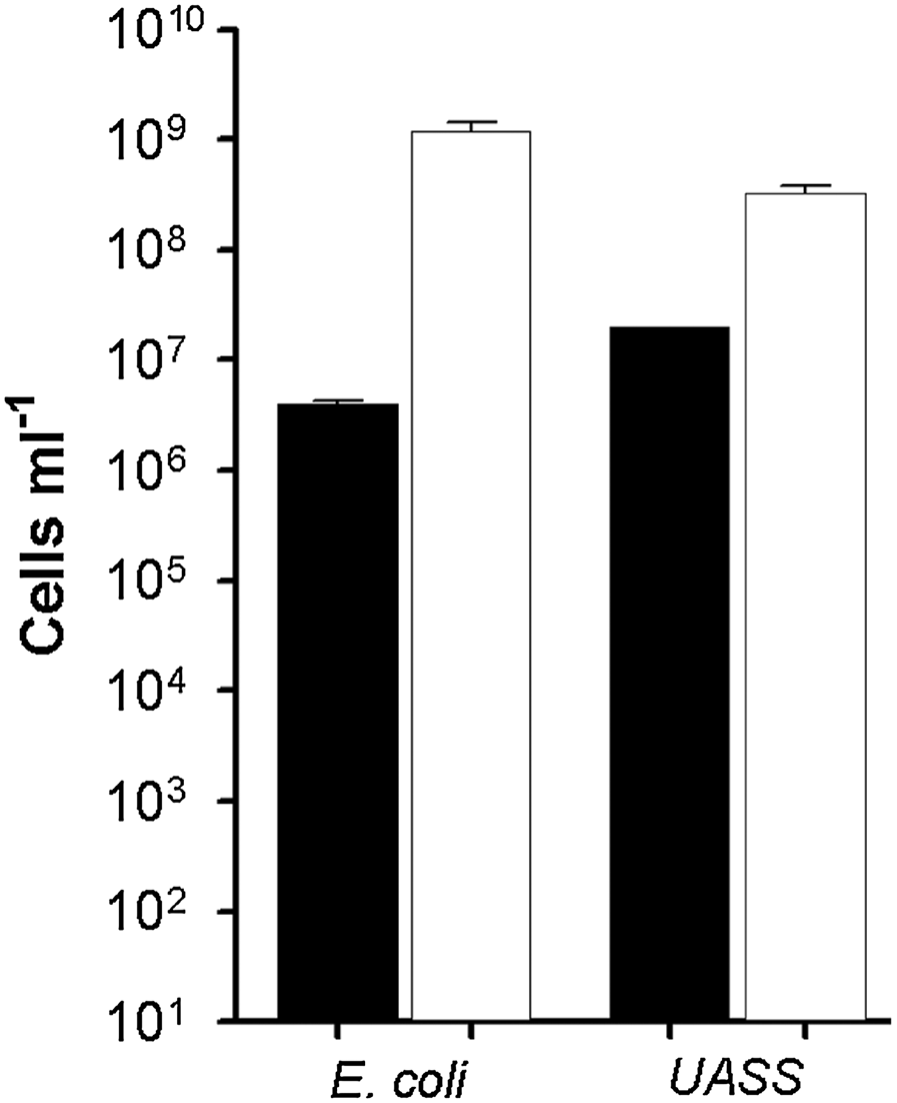
**Influence of dehydration and associated centrifugation steps prior to FISH hybridization on cell counts.** The bar charts represented the cell counts for *E. coli* cultures and UASS biogas reactor samples with (black bars) and without (white bars) dehydration steps during the FISH procedure. All samples were pretreated with purification procedure 1-C2-S2-H1-F2. Error bars resulted from nine different measurements with Coulter Counter. In case of UASS sample with dehydration step (black) only three measurements were conducted.

The application of conventional FISH protocols according to Amann et al. (1990) [11], Wallner et al. (1993) [18], and Grzonka (2008) [30] for Flow-FISH technique resulted in high cell losses due to the centrifugation steps as part of the dehydration steps. With *E. coli* cultures, performing dehydration steps reduced the detected cell number by two to three log units (Figure 4). For UASS reactor samples a lower cell loss of about one log unit was determined after performing dehydration steps (Figure 4). Hence, to avoid high cell losses, dehydration and most centrifugation steps were abandoned in the new optimized FISH protocol.

**Figure 5:**
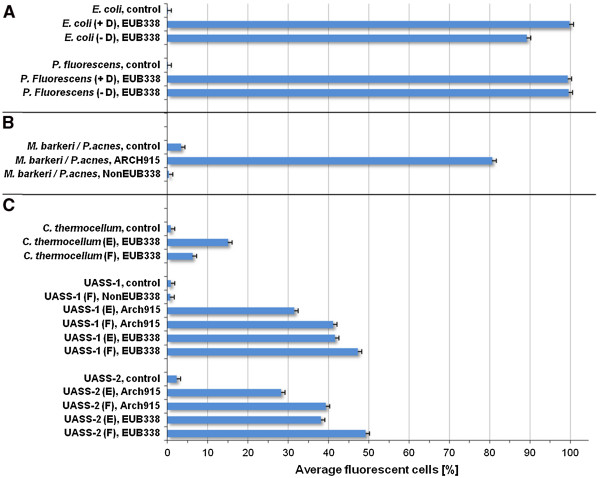
**Establishment of Flow-FISH protocol.** The average percentage of cells hybridized with AlexaFluor488 labeled oligonucleotide probes for bacteria (EUB338), archaea (ARCH915), and the nonsense probe NonEUB338 was determined by flow cytometry at 488 nm excitation: **(A)** Effect of dehydration on FISH hybridization rate using pure cultures of *E. coli* and *Pseudomonas fluorescens*; +D = with dehydration steps before hybridization, -D = without dehydration steps before hybridization. **(B)** Proof of possible cross hybridization effects using mixed culture of *Methanosarcina barkeri* (archaea) and *Propionibacterium acne* (bacteria). **(C)** Influence of fixative on FISH hybridization rate using a pure culture of *Clostridium thermocellum* and two independent samples of a mesophilic UASS biogas reactor (UASS-1 and UASS-2); F = sample was fixed with 3.7% formaldehyde, E = sample was fixed with 50.0% ethanol. For all experiments a control sample without any FISH probe was applied to detect possible cell autofluorescence. All samples were pretreated with purification procedure 1-C2-S2-H1-F2. Error bars resulted from three different measurements.

In this study, the effect of dehydration or non-dehydration, respectively, on the hybridization rate of FISH probe EUB338 was determined with two pure cultures, *E. coli* and *P. fluorescens* (Figure 5A). In case of *P. fluorescens* no effect of dehydration on success of FISH was obvious, whereas in case of *E. coli*, the Flow-FISH protocol including dehydration steps showed a quite higher hybridization rate. For purified UASS biogas reactor samples no effect of omitted or performed dehydration on the hybridization rates was detected. To avoid false positive fluorescence signals caused by cell autofluorescence during measurement by flow cytometer, hybridizations without probes were performed [9]. These negative controls resulted in no fluorescence signals indicating the absence of microbial autofluorescence (Figure 5A). The ethanol dehydration could support the cell membrane permeability of some prokaryotes for FISH probes resulting in a higher hybridization rate. However, this effect may differ from organism to organism. Therefore, every sample needs to be controlled for dehydration effects on cell counts and hybridization rates, especially in case of mixed cultures or environmental samples.

For the verification of a possible cross hybridization of the specific FISH probe with non-target individuals the NonEUB338 probe was used standardly. This nonsense probe is reverse complementary to EUB338 probe and has no known 16S rRNA target. The test was conducted using a mixed culture of *Methanosarcina barkeri* (Archaea) and *Propionibacterium acne* (Bacteria) (Figure 5B). Whereas hybridization of *M. barkeri* / *P. acne* mixed culture using the probe ARCH915 resulted in a high hybridization rate of about 80% of all cells, no fluorescence signal was determined with NonEUB338. This indicates that the chosen hybridization conditions did not promote any cross hybridization of archaeal FISH probe with bacterial cells in this culture. Furthermore, FISH without any probe was performed with the same sample to evaluate possible background fluorescence because it is well known that *P. acne* exposed a low red autofluorescence [33,34]. As expected, in this experiment the control sample of the mixed culture showed minor background fluorescence (Figure 5B).

Another factor influencing the result of Flow-FISH is the choice of the fixative for the necessary cell fixation immediately after sampling. Because most environmental samples include both Gram-negative and Gram-positive prokaryotes, it is generally recommended to prepare both, formaldehyde- as well as ethanol-fixed samples. In this study, both fixation procedures were carried out with pure cultures of *C. thermocellum*, as a typical representative for Gram-positive prokaryotes in biogas reactors, as well as samples of UASS biogas reactor. In case of *C. thermocellum,* the fixation with 50% ethanol led to an increased hybridization rate when using the bacteria universal probe EUB338 (Figure 5C). In contrast, in case of the UASS reactor sample, the fixation with 3.7% formaldehyde resulted in better hybridization rates than obtained after ethanol fixation regardless of which FISH probe was applied. The sum of archaea and bacteria cell counts in formaldehyde fixed samples achieved about 90% of total cell counts determined by flow cytometry (Figure 5C). Interestingly, the percentage of archaea, i.e. about 40% of total cell counts, is relatively high compared with previously published results [4,23,35,36]. On the other hand, Fredriksson and co-worker (2012) [3] studied the diversity and the dynamic of archaea community in different compartments of an activated sludge wastewater treatment plant (WWTP) and determined an average percentage of archaea cells up to 75% using confocal laser scanning microscopy. Because the negative control hybridizations with probe NonEUB388 and the subsequent measurements in flow cytometer did not show any fluorescent cells, the absence of cross hybridization effects for UASS samples is indicated (Figure 5C). The low hybridization rates observed for bacteria in UASS samples and *C. thermocellum* could be caused by a lower metabolic activity of parts of these cells. Microorganisms in the environment often do not grow at their optimal rate and could show different metabolically stages: active, inactive, starved, and dormant. Generally, microbial cells with metabolic activity have a sufficient number of 16S rRNA molecules which were usually used as targets for fluorescently labeled FISH probes. In consequence, a sufficient number of 16S rRNA molecules is required for strong fluorescence signals in flow cytometry or fluorescence microscopy, respectively [7,8,37].

### Determination of the microbial metabolic state

Because of the low hybridization rate partially observed for some samples (Figure 5), the metabolic cell activity was determined by examination of dehydrogenase activity visualized by 5-Cyano-2,3-ditolyl tetrazolium chloride (CTC) reduction in microbial cells. CTC is reduced to CTC formazan by electron transfer through respiratory activity and accumulates as red fluorescent crystals inside the cell [38-40]. This enables the detection of active cells by flow cytometry as well as by fluorescence microscopy. Therefore, a regular sampling within 24 h from the UASS biogas reactor as well as growth series of *E. coli* and *C. thermocellum* were performed.

At anaerobic conditions an abiotical reduction of CTC is possible [38]. Hence, inactivated samples from the UASS reactor as well as *E. coli* and *C. thermocellum* cultures were used as negative controls to exclude possible false positive fluorescence signals. No fluorescence signals could be detected from any inactivated samples after CTC incubation indicating that no abiotical reduction of CTC occurred at the apparent experimental conditions (data not shown).

**Figure 6:**
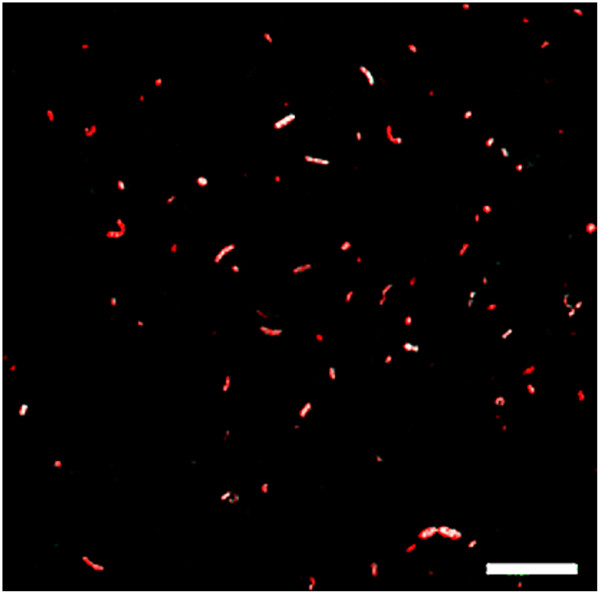
**Evaluation of CTC treated UASS sample 3 h after feeding with wheat straw by confocal laser scanning microscopy.** Total cell counts were determined by counting SYTO60 stained cells (red color). CTC-formazan fluorescence is shown in blue (outside cells) or white (inside cells). Micrographs are overlays of sequential scans. Scale bar equals 10 μm.

The evaluation of UASS samples after CTC incubation was difficult. Because it could not be ruled out that the CTC formazan crystals will be washed out of the cells during purification procedure as described above, we decided to pass on the sample pretreatment. Hence, measurement by flow cytometry could not be conducted and cell counts in UASS samples were estimated by microscopic field analysis. Because of background fluorescence of unpurified UASS samples a reliable quantification of total cell count as well as of CTC-formazan positive cells was not possible. In general, the activity of cells in UASS reactor samples was low according to CTC-formazan staining. An example of analyzed UASS reactor sample 3 h after supply with wheat straw as substrate is shown in Figure 6. About 43% to 60% of total cells showed a positive CTC-formazan fluorescence signal regardless of the time of sampling indicating active cells which were in consequence detectable by Flow-FISH.

**Figure 7:**
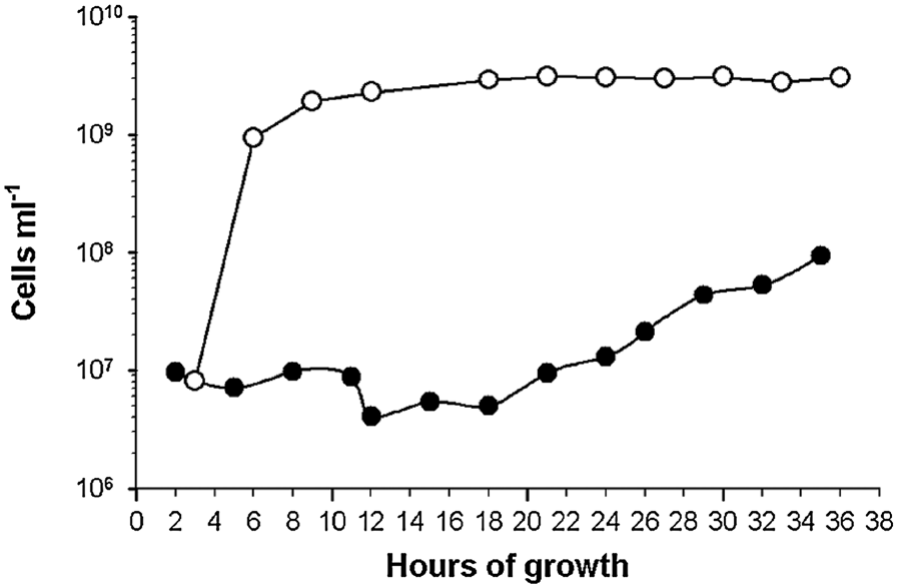
**Growth series.** Cell counts of *E. coli (−○-)* and *C. thermocellum (−●-)* evaluated every 3 h over a growth period of 36 h. At each data point cells were tested for cell activity by CTC incubation (see Figure 8). Cell counts were determined in triplicate by Coulter Counter.

**Figure 8:**
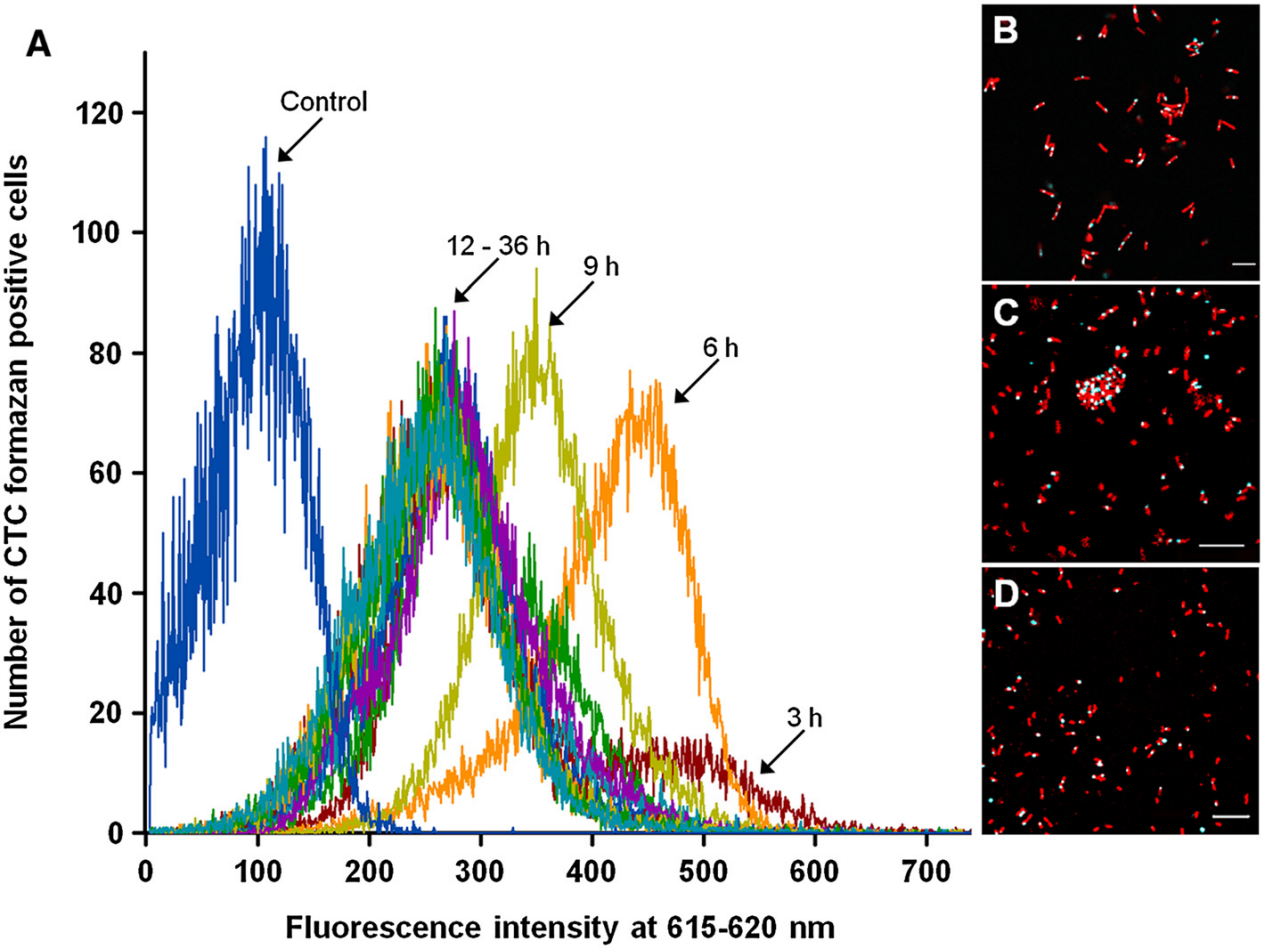
**Dehydrogenase activity in *E. coli *cultures determined by CTC treatment.** Samples were taken every 3 h over a total growth period of 36 h. An untreated *E. coli* culture was used as control. Fluorescence emissions were determined by flow cytometry **(A)** and by confocal laser scanning microscopy **(B-D)**. Images **B-D** show CTC treated *E. coli* cells after growth of 3 h **(B)**, 6 h **(C)**, and 9 h **(D)**. Total cell counts were determined by counting SYTO60 stained cells (red color). CTC-formazan fluorescence is shown in blue (outside cells) or white (inside cells). Micrographs are overlays of sequential scans. Scale bar equals 10 μm.

Because of the difficult conditions, as described above, for the evaluation of the metabolic activity of microorganisms in UASS reactor samples, this experiment was also applied for growth series of *E. coli* and *C. thermocellum* pure cultures. Photometric analyses of the growth state of pure cultures resulted in a typical growth curve of *E. coli* with an exponential growth phase in the first 12 h followed by a long stationary phase (Figure 7). The results of CTC incubation determined by flow cytometry showed that *E. coli* cells were highly active after a growth time of 3 h (Figure 8A). This was also verified by confocal laser scanning microscopy (Figure 8B-C). At growth time of 3 h the highest fluorescence signals of CTC-formazan were determined whereas the lowest cell number of *E. coli* was measured (Figures 7 and 8). Furthermore, flow cytometry has shown that the cell number of *E. coli* pure culture increased during the first 12 h. Overall, the cell number increased with increasing growth time but fluorescence signals of cells decreased simultaneously (Figures 7 and 8A-C) which indicates that the cells reduced their metabolic activity during growth. In consequence the number of ribosomes and 16S rRNA molecules in these cells was also decreased. DeLong and co-workers (1989) [6] have shown that the fluorescence signal intensity is directly related to the physiological state of the cells. However, other studies have shown that slowly growing bacteria can possess high numbers of ribosomes or, in contrast, highly active microorganisms can have low numbers of ribosomes [30,37,41,42].

In contrast, the growth curve from *C. thermocellum* showed a long lag phase of approximately 20 h followed by a weak exponential growth phase (Figure 7). Due to the limitation on 36 h, the end of the exponential growth phase and the beginning of the stationary growth phase could not be determined during this experiment. Furthermore, CTC-formazan fluorescence signals could only be determined after 22 h growth time. However, fluorescence signals before a growth time of 22 h were quite low (microscopic data not shown). Thus, the low hybridization rate of *C. thermocellum* detected by Flow-FISH could have been caused by a low metabolic cell activity and, consequently, by a low 16S rRNA concentration in the cells. The results of both experiments are in accordance to further studies [6-8,37].

## Conclusions

In this study, a protocol for purification of high heterogenic liquid samples from biogas reactors for the analysis of microbial community by flow cytometry was successfully developed. Furthermore, a Flow-FISH protocol was established to detect process-relevant active microorganisms in biogas reactor samples.

The developed purification procedure (1-C2-S2-H1-F2) is based on the treatment with sodium hexametaphosphate and ultrasound treatment with a final filtration step. We demonstrated that cell aggregates could successfully be suspended and cells were successfully removed from organic or inorganic particles and that these particles were eliminated from the samples using this purification procedure. Moreover, the cell loss due to purification was negligible.

Furthermore, a modified Flow-FISH protocol for analysis of microbial community biogas reactors was successfully adapted in this study. The waiver of dehydration steps decreased the cell loss during procedure but this may also decrease the hybridization rate of some bacteria species. Therefore, the benefit on cell counts by omission of dehydration should be decided from case to case. However, we have shown that the applied Flow-FISH protocol did not allow cross hybridization determined by use of the nonsense probe NonEUB338. In addition, false positive fluorescence signals caused by background fluorescence or autofluorescence of microorganisms were also excluded by using control hybridizations without any FISH probes.

The new developed purification technique in combination with a modified Flow-FISH protocol described in this paper enables for the first time a high throughput analysis of microbial communities in heterogenic samples from biogas reactors focused on the detection of process-relevant, metabolically active microorganisms.

## Methods

### Cultivation of pure cell cultures

Pure cultures of *Escherichia coli* (DSM 1116), *Pseudomonas fluorescens* (DSM 50090), and *Clostridium thermocellum* (DSM 1237) as well as a mixed culture of *Methanosarcina barkeri* (DSM800) and *Propionibacterium acne* (DSM1897) were cultivated under defined conditions as control samples. Therefore, cryo beads of *E. coli* and *P. fluorescens* were pre-cultivated over night at 37°C (*E. coli*) or 30°C (*P. fluorescens*) in filtrated Nutrient Broth (NB) medium. For this pre-culture, approx. 10^6^ cells per ml were used to inoculate 100 ml fresh and NB medium. These cultures were incubated for 10 h at the respective optimal growth temperature to obtain the working culture. *C. thermocellum* cells were cultivated in GS2 nutrient solution [43] at anaerobic conditions at 55°C for 30 h. *M. barkeri* and *P. acne* were cultivated in mixed culture in DSM medium 120 (Leibniz Institute DSMZ, German Collection of Microorganisms and Cell Cultures, Germany) at anaerobic conditions at 37°C for 48 h. All culture media were sterilized by autoclaving process before use.

### Operation and sampling of the biogas reactor

The design and operation of the upflow anaerobic solid state (UASS) reactor connected with a downstream anaerobic filter (AF) reactor was described in detail by Pohl et al. (2012) [44]. For this study, chopped wheat straw was used as substrate at an organic loading rate (OLR) of 2.5 g volatile substances (VS) per liter and day. The UASS reactor was operated at mesophilic temperatures (37°C). Two liquid samples were taken from the effluent of the UASS reactor at various times (hereafter referred to as UASS-1 und UASS-2). Samples were processed immediately after sampling for further analyses.

### Sample fixation

Sample fixation was carried out immediately after sampling according to a protocol after Kepner and Pratt (1994) [45]. Therefore, 10 ml of pure cultures or liquid samples from the UASS reactor, respectively, were fixed with 30 ml of a 3.7% formaldehyde solution (diluted in 1× PBS pH 7.4) for 4 h at 4°C. After fixation, the samples were centrifuged at 8,000 × *g* for 20 min at room temperature (RT). The supernatant was discarded and the pellet was washed twice in 1× PBS using same centrifugation conditions as before. The 1× PBS was prepared of 140 mM NaCl, 10 mM Na_2_HPO_4_, 2.7 mM KCl, and 1.8 mM KH_2_PO_4_. The pH was adjusted to 7.4 with HCl (all reagents were provided by Carl Roth GmbH & Co. KG, Germany). After washing the pellet was re-suspended in 5 ml 1× PBS, mixed with 5 ml 96% ethanol p.a. and stored until further use at −20°C.

Alternatively, a fixation with 50% ethanol (diluted in 1× PBS pH 7.4) was performed for Gram-positive prokaryotes. In this case, the samples were centrifuged at 8,000 × *g* for 20 min. The pellets were re-suspended in 5 ml 1× PBS, mixed with 5 ml 96% ethanol p.a. and stored until further use at −20°C.

### Sample pre-treatment for Flow-FISH analyses

Six different pre-treatment techniques for sample purification taken from the recent literature (in the following denominated as procedure 1 to procedure 6) were tested on both, pure cultures and UASS biogas reactor samples. An overview about all pre-treatment procedures and their modifications is given in Table 1.

The following modifications of these procedures were conducted: (a) varying concentrations of detergents (Table 1, index C), (b) ultrasound treatment at varying intensities (Table 1, index S), (c) without and with homogenization at varying intensities (Table 1, index H), and (d) with or without filtration (Table 1, index F).

For procedure 1, 10 ml of fixed sample was centrifuged at 8,000 × *g* for 20 min at room temperature. For procedures 2–6, a similar volume was centrifuged at 15,000 × g for 5 min at room temperature. Afterwards, all preparations were washed once with 1× PBS (pH 7.4) to remove ethanol. The solid residues were re-suspended according to the respective literature. All applications were carried out in triplicates. In the following, purification procedure 1 is described in detail because this procedure is the optimized pre-treatment method for Flow-FISH, while the other pre-treatment techniques were carried out as published previously (Table 1). All applied modifications are described in Table 1.

Procedure 1 modified after Singh-Verma [22] and Bakken [24,26]: The cell pellet was washed with sterile 1× PBS (pH 7.4). After centrifugation at 8,000 × *g* for 20 min the cell pellet was re-suspended in 10 ml sterile 0.5% sodium hexametaphosphate (pH 8.5, Sigma-Aldrich, Germany). After 10 min of incubation the sample was sonicated at 65 W for 1 min (Sonoplus GW2070, Bandelin, Berlin, Germany). A centrifugation step at 650 × *g* for 2 min was conducted to separate microorganisms from organic or inorganic particles in the sample. The supernatant containing free cells was transferred in a sterile tube for further application. The residual cell pellet was re-suspended in 10 ml sterile 0.5% sodium hexametaphosphate (pH 8.5) and incubated for 10 min followed by a further ultrasonic treatment and centrifugation step. The sodium hexametaphosphate incubation step, the ultrasound step, and the centrifugation step were repeated up to five times depending on sample consistence. After five repetitions, the remaining pellet should consist mainly of organic and inorganic material and a negligible quantity of free microbial cells. The supernatants containing free microbial cells were pooled in a sterile tube. The cells were collected by centrifugations at 8,000 × *g* for 20 min. The supernatant was discarded and the pelleted cells were re-suspended in 10 ml 1× PBS (pH 7.4). Afterwards, a vacuum filtration of the sample using a sterile filter with 12–15 μm pore size was conducted. The filter was washed once with 40 ml 1× PBS (pH 7.4). Subsequently, the filtrate was centrifuged at 8,000 × *g* for 20 min. The supernatant was discarded, and the pellet was re-suspended in 10 ml of 1× PBS (pH 7.4) and used for the Flow-FISH analysis. In addition, the residues on the filter were collected described as following: to re-suspend particles and cells the filter was transferred into a 50 ml tube and incubated in 9 ml 1× PBS (pH 7.4) at room temperature for 20 min with slow rotation. After incubation, the filter was carefully rinsed off with 1 ml 1× PBS (pH 7.4). The residues on the filter were subsequently used for the microscopic verification of purification success.

All samples purified by the six procedures were stored at 4°C no longer than 12 h until further processing.

### Verification of purification procedures

One important criterion for a purification method is a minimized loss of cells. Unfortunately, cell densities of untreated biogas reactor samples could not be calculated by particle counting due to interfering particles and cell aggregates. Hence, pure cultures of *E. coli* were used for determination of cell losses during the purification procedures. Cell counts were determined in triplicates by Coulter Counter (Multisizer™ 3 Coulter Counter®, Beckman Coulter, Germany). Each triplicate was measured three times and the standard deviation of the nine measurements was calculated. Measurements were carried out with a 50 μm capillary, and the measurement volume was 50 μl. To determine the particle number and size within the electrolyte solution (‘background control’), the electrolyte was measured without addition of any microorganisms.

For the verification of the purification success in terms of cell aggregates disbandment and detachment of microorganisms from particles, the washed pellets, the supernatants, and the residues on the filter were visually evaluated by fluorescence microscopy. For microscopic analyses 10 μl of residues on the filter, pellet samples, and supernatants each diluted 1:500 in sterile water were coated on separate wells of a 10-well-slide in triplicates. After drying the samples at 40°C the antifading reagent Citifluor A1 (PLANO GmbH, Wetzlar, Germany) was added to coat each well and 0.2 μl of a 20 μg ml^-1^ stock solution of 4’,6-diamidino-2-phenylindole (DAPI) were carefully injected into the Citifluor A1 drop. The size of cell aggregates was determined by microscopic field analyses using an ocular micrometer at 630× magnification. Five randomly chosen microscopic fields from each sample were analyzed in terms of the sizes of cell aggregates, the presence of organic and inorganic particles, and their microbiological growth. One microscopic field comprised the total area of 144 μm^2^ and was divided into 10 × 10 sub-fields of 5.76 μm^2^ each. All microscopic analyses were conducted with a Nikon Optiphot-2 microscope (Nikon, Duesseldorf, Germany) fitted with a DAPI AMCA filter tube or with an Olympus BX51 fluorescence microscope (Olympus GmbH, Hamburg, Germany) fitted with a U-MWU2 filter module.

### Fluorescence in situ hybridization (FISH)

FISH was carried out with domain specific probes EUB338 (5′-GCTGCCTCCCGTAGGAGT-3′) [46] and ARCH915 (5′-GTGCTCCCCCGCCAATTCCT-3′) [47] for the detection of bacteria and archaea, respectively. For the detection of undesired cross hybridization with non-target microorganisms the nonsense probe NonEUB338 (5′-ACTCCTACGGGAGGCAGC-3′) [20] was used. Furthermore, negative controls without the addition of probes were performed to determine autofluorescence effects. All FISH probes were labeled with fluorescent dye Alexa488 and were manufactured by Eurofins MWG GmbH (Ebersberg, Germany). Flow-FISH was carried out in triplicates which were each analyzed three times by flow cytometry. Based on these in total nine measurements an average with a standard deviation was calculated.

The modified protocol for Flow-FISH of biogas reactor samples established in this study consists of following steps: 250 μl fixed sample was centrifuged at 8,000 × *g* for 20 min. All centrifugation steps were conducted at room temperature. The supernatant was discarded, and the pellet was re-suspended in 221 μl of 46°C preheated hybridization buffer (0.9 M NaCl, 20 mM Tris/HCl (pH 7.2), 0.1% SDS and 50% formamide) and 21 μl of the FISH probe (50 ng μl^-1^). During incubation at 46°C for 2 h, the sample was repeatedly inverted. A centrifugation step at 8,000 × *g* for 20 min ensured the pelleting of microbial cells. The cell pellet was washed twice with 500 μl 0.05 M PBS pH 7.0 using the same centrifugation conditions as before. The phosphate buffered saline (PBS) was prepared of 137 mM NaCl, 2.7 mM KCl, 40.6 mM Na_2_HPO_4_, and 7.1 mM KH_2_PO_4_. The pH was adjusted to 7.0 with HCl and the buffer was finally filtered with a 0.2 μm membrane filter.

For comparison, the following conventional FISH protocol according to Amann et al. (1990) [11], Wallner et al. (1993) [18], and Grzonka (2008) [30] was also performed: 1 ml fixed sample was centrifuged at 8,000 × *g* for 20 min. The pellet was dehydrated stepwise in 1 ml 50%, 80% and 96% ethanol for 3 min each. After each ethanolic treatment a centrifugation at 8,000 × *g* for 20 min was conducted. After completed dehydration the pellet was re-suspended in 46°C preheated hybridization buffer (0.9 M NaCl, 20 mM Tris/HCl (pH 7.2), 0.1% SDS, and 50% formamide) containing FISH probe with an end concentration of 5 ng per μl. The hybridization was carried out in the dark for 2 h at 46°C in a water bath with occasional inverting. To remove hybridization buffer and non-bound probes the samples were centrifuged at 8,000 × *g* for 20 min and washed with 0.05 M PBS (pH 7.0). After further centrifugation at 8,000 × *g* for 20 min, the pellet was re-suspended in 0.05 M PBS (pH 7.0) to obtain a cell concentration of approximately 10^6^ cells per ml suited for subsequent flow cytometric analysis.

### Flow cytometry

For flow cytometry, a Cytomics FC500 (Beckman Coulter, Deutschland) or a CyFlow ML (Partec, Deutschland) platform were used. In case of the Cytomics FC500, the field stop was set on 1 - 19°, and the discriminator to reduce background noise was set on the side scatter (SS = 2). For all platforms, the fluorescence of the probes was excited with a laser at a wavelength of 488 nm and the emission was measured using a photomultiplier and a band pass filter of 525 ± 25 nm (Cytomics FC500) or 536 ± 40 nm (CyFlow ML). Samples without probes were measured to adjust the voltage of the photomultiplier to avoid the measurement of autofluorescent cells. A total of 10,000 (Cytomics FC500) or 100,000 (CyFlowML) events were collected in all runs.

### Determination of the microbial metabolic activity

The low hybridization rate for bacteria in the UASS biogas reactor samples indicated that not all bacteria possessed the high metabolic activity essential for a strong fluorescence signal. Hence, the metabolic activity of the microbial cells needed to be evaluated. Therefore, the dehydrogenase activity was determined by incubation with 5*-*cyano*-*2*,*3*-*ditolyl tetrazolium chloride (CTC) according to the protocol of Preuss and Hupfer (1998) [48] based on a modified protocol of Rodriguez and co-workers (1992) [49].

This assay was tested with growth series of pure cultures of *E. coli* and *C. thermocellum* as well as with a time series of UASS reactor samples. Samples of the *E. coli* and *C. thermocellum* culture were taken every 3 h between 3 and 36 h of growth. Samples from UASS biogas reactor were taken 1, 3, 5, 7, 9, 20, and 22 h after last feeding.

From each sample, triplicates of 1 ml were inoculated with 100 μl of a 0.16% CTC solution and incubated at 37°C for 60 min with constant shaking at 450 rpm (Thermomixer comfort, Eppendorf, Germany) and at dark conditions. As negative controls, 1 ml triplicates of each sample were inactivated for 20 min at 95°C with constant shaking at 700 rpm (Thermomixer comfort, Eppendorf, Germany) and treated as described above. The CTC reaction was stopped by adding 10 μl 37% formaldehyde. From each sample, a dilution series (100-, 500- and 1000-fold) was performed with sterile water.

For microscopic quantification of active and inactive cells 10-well-slides were coated with an aqueous solution of 0.1% gelatin and 0.01% CrK (SO_4_). 10 μl of each sample dilution was added to the wells and dried by air at room temperature. Subsequently, 5 μl antifading reagent Citifluor A1 (PLANO GmbH, Wetzlar, Germany) was added to coat each well, and 0.2 μl of a 5 μM stock solution of SYTO60 were carefully injected into this drop. After 20 min incubation the samples were ready to use for microscopic analysis by confocal laser scanning microscopy (TCS SP5 II, Leica Microsystems, Germany) using LAS AF Leica software. Following system settings were used: scan mode xyz - pinhole 1.50 airy, Acusto-Optical Tunable Filter (AOTF) 514 nm (10%), AOTF 633 nm (10%); sequential scan settings for SYTO60 - 633 nm, photo multiplier tubes (PMT) 650–770 nm; sequential scan settings for CTC - AOTF 514 nm, PMT 570–640 nm. The settings for picture size, gain, and offset were varied during the experiment to reach best image resolution and fluorescence signal strength.

In addition, samples were analyzed by flow cytometry. The Cytomics FC500 platform was used with following settings: excitation of CTC fluorescence at 488 nm, photomultiplier wavelength 615–620 nm. All further details were as given above.

## Competing interests

The authors declare that they have no competing interests.

## Authors’ contributions

EN and AF conceived the experimental design on Flow-FISH and carried out the experiments, evaluated the results, and drafted the manuscript. EN conceived the experimental design on sample pretreatment. KH collected and provided the biogas reactor samples and helped to draft the manuscript. MK, OS, and JM participated in the design of the study and provided substantial expertise on microbial community structure in biogas reactors, flow cytometry analysis, and performance and processes of UASS biogas reactor, respectively. All authors contributed to writing the manuscript and read and approved the final version.
